# Calcification in Werner syndrome associated with lymphatic vessels aging

**DOI:** 10.18632/aging.203789

**Published:** 2021-12-27

**Authors:** Hideyuki Ogata, Shinsuke Akita, Sanae Ikehara, Kazuhiko Azuma, Takashi Yamaguchi, Maihulan Maimaiti, Yoshiro Maezawa, Yoshitaka Kubota, Koutaro Yokote, Nobuyuki Mitsukawa, Yuzuru Ikehara

**Affiliations:** 1 Department of Plastic, Reconstructive, and Aesthetic Surgery, Graduate School of Medicine, Chiba University, Chiba, Japan; 2 Department of Pathology, Graduate School of Medicine, Chiba University, Chiba, Japan; 3 Cellular and Molecular Biotechnology Research Institute, National Institute of Advanced Industrial Science and Technology (AIST), Tsukuba, Japan; 4 Department of Endocrinology, Hematology and Gerontology, Graduate School of Medicine, Chiba University, Chiba, Japan; 5 Division of Diabetes, Metabolism and Endocrinology, Chiba University Hospital, Chiba, Japan

**Keywords:** Werner syndrome, calcification, skin ulcer, lymphatic vessel, endoplasmic reticulum stress

## Abstract

In addition to the symptoms of aging, the main symptoms in Werner syndrome (WS), a hereditary premature aging disease, include calcification of subcutaneous tissue with solid pain and refractory skin ulcers. However, the mechanism of calcification in WS remains unclear. In this study, the histological analysis of the skin around the ulcer with calcification revealed an accumulation of calcium phosphate in the lymphatic vessels. Moreover, the morphological comparison with the lymphatic vessels in PAD patients with chronic skin ulcers demonstrated the ongoing lymphatic remodeling in WS patients because of the narrow luminal cross-sectional area (LA) of the lymphatic vessels but the increment of lymphatic microvessels density (MLVD). Additionally, fluorescence immunohistochemical analysis presented the cytoplasmic distribution and the accumulation of WRN proteins in endothelial cells on remodeling lymphatic vessels. In summary, these results point out a relationship between calcification in lymphatic vessels and the remodeling of lymphatic vessels and suggest the significance of the accumulation of WRN mutant proteins as an age-related change in WS patients. Thus, cytoplasmic accumulation of WRN protein can be an indicator of the decreasing drainage function of the lymphatic vessels and the increased risk of skin ulcers and calcification in the lymphatic vessels.

## INTRODUCTION

Werner syndrome (WS) is a rare autosomal recessive disorder in which a variety of age-related diseases appear at an early age [1, 2]. The cause of this disease is a mutation of the WRN gene located in the WS locus at 8 p 12 [3]. WRN proteins have helicase and exonuclease activities [4–6] and appear to be involved in DNA repair [7].

Typical age-related disorders in WS include arteriosclerosis, malignant neoplasms, type 2 diabetes mellitus, dyslipidemia, osteoporosis, and ocular infections. In addition, calcification of the skin and soft tissues is associated with severe pain and skin ulceration, which is often challenging to treat and, therefore, impairs patients' quality of life [8]. It is noteworthy that calcification of the skin and soft tissues is a characteristic feature of WS: [1, 2, 9–12] because it is often painful enough to lead to a physician's visit [9–13]. Moreover, calcification is used as an indicator for an early diagnosis of WS in less clear cases of age-related diseases [13] because calcification in WS presents on x-rays with characteristic flame-like shadows are pathognomonic findings and differ from the calcification patterns in other disorders [12].

Our previous study showed that overexpression of Na-Pi cotransporter (Pit-1) in dermal fibroblasts in WS contributed to deposit calcium *in vitro* as the cause of calcification [13], while the mechanism of calcification at the tissue level *in vivo* has not yet been shown despite the essential findings to diagnosis for WS in current guidelines.

In the present study, we examined the pathological features of skin ulcer lesions with the characteristic calcification of WS. We observed that the calcification in patients with WS spread along the lymphatic vessels on the boundary between the dermis and subcutaneous tissue. and that the proliferation of lymphatic vessels with a narrow lumen was prominent in the interstitial tissue with less calcification. Because these findings strongly suggested a relationship with the radiographic appearance of flame-like shadows, we examined the expression of WRN protein in lymphatic endothelial cells (LECs) by immunohistochemical staining to investigate the relationship between soft tissue calcification and lymphatic vessel function in WS.

## RESULTS

### WS causes calcification in the lymphatic vessels of the skin around the ulcer

**Figure 1:**
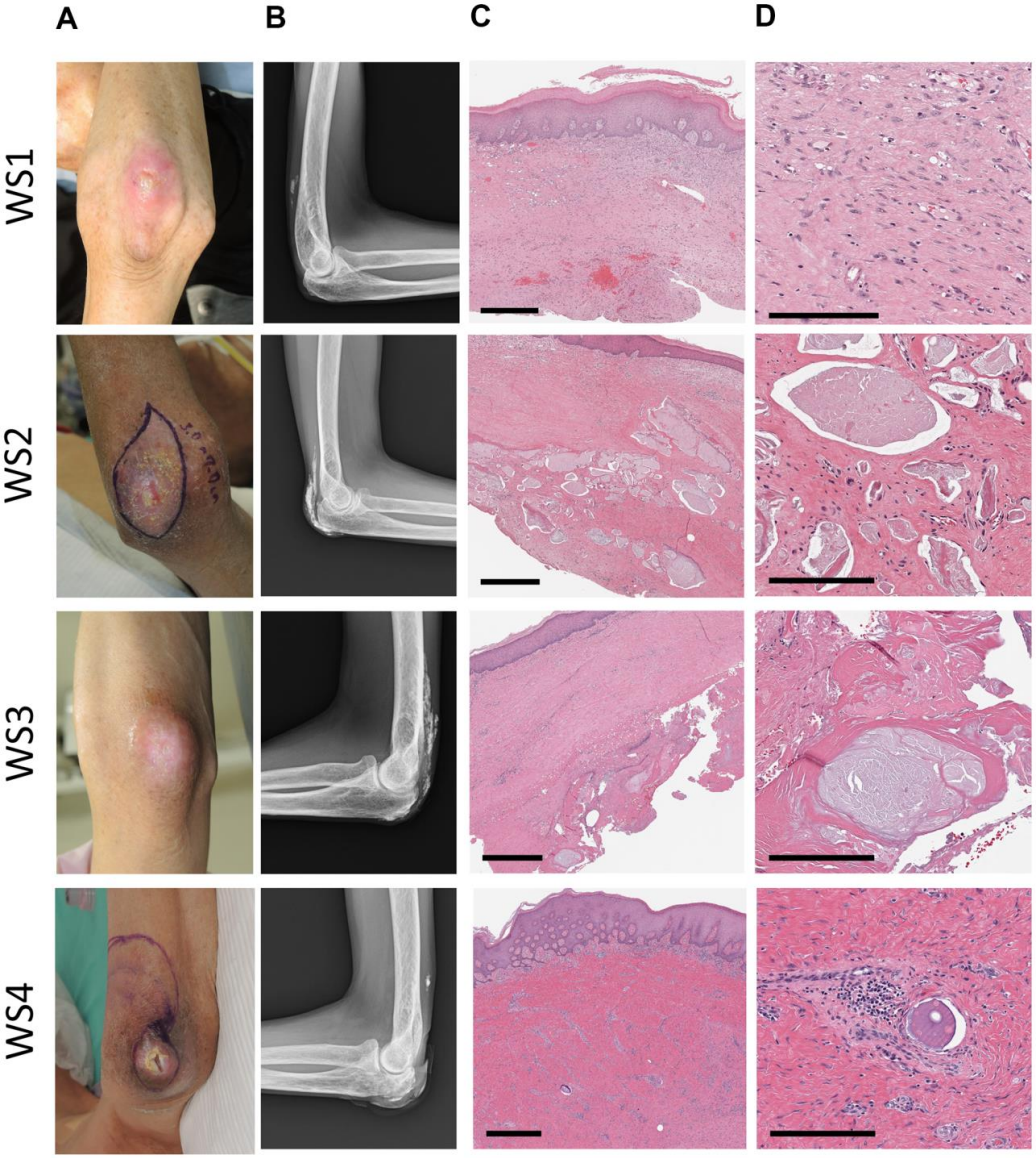
**Clinical images of patients.** A series of photographs (**A**) of elbow ulcers in four patients with Werner's syndrome (WS1-4), radiographs (**B**) of the same area, and HE stained tissue (**C**, **D**) of the skin around the ulcers. WS1: 62-year-old male, WS2: 48-year-old male, WS3: 50-year-old female, WS4: 57-year-old male. (**A**) The skin around the ulcer is sclerotic and atrophic. (**B**) Various degrees of calcification were observed in the soft tissues around the elbow joint. In WS2 and WS3, it becomes a shadow of the flame state. (**C**, **D**) Strong fibrosis of the dermis (all cases) and calcification within the luminal structures (WS2, 3, 4). Scale bars are 700 μm (**C**) and 200 μm (**D**).

**Figure 2:**
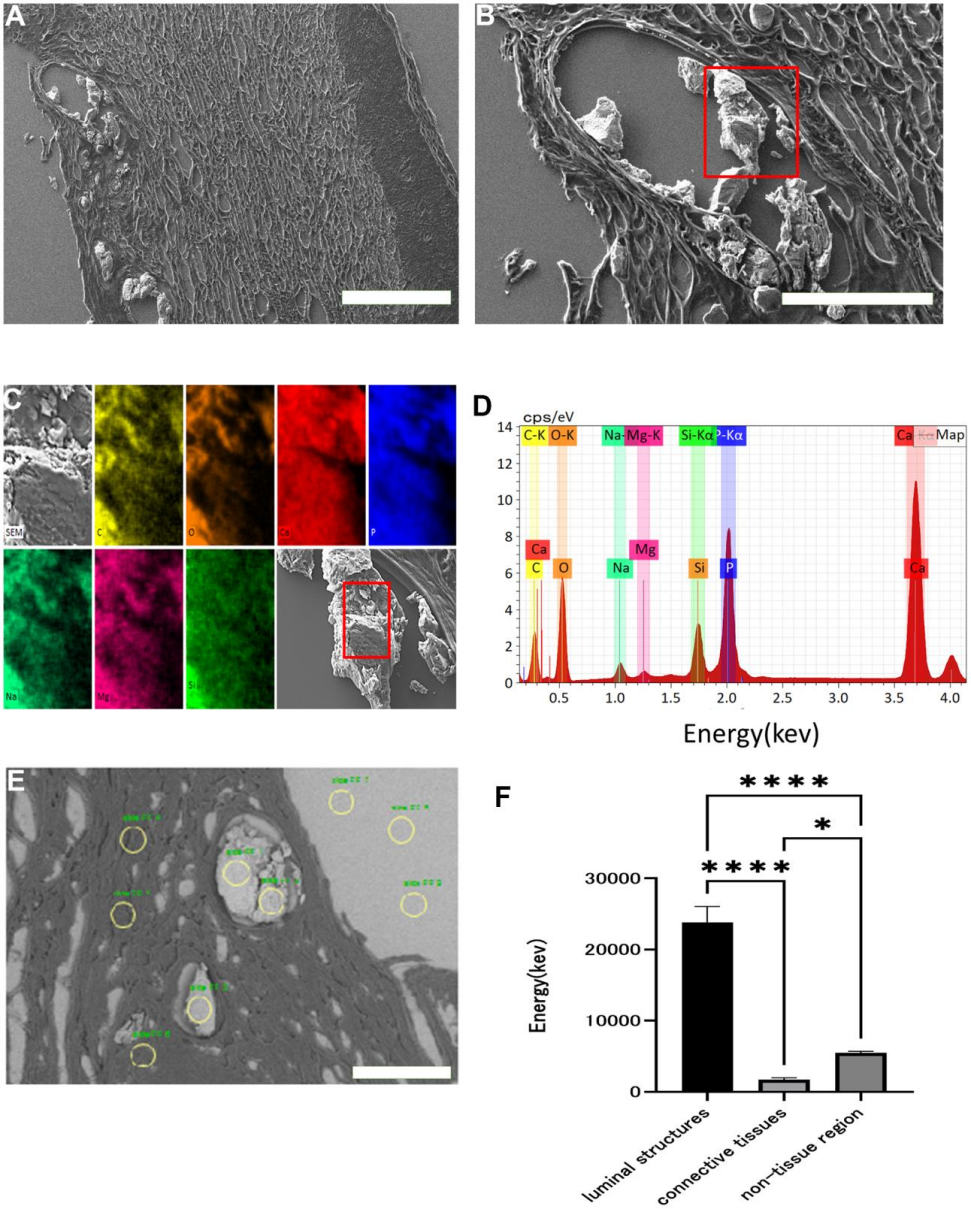
**Scanning electron microscopy images and EDX analysis.** Scanning electron micrograph of skin around an ulcer on a patient's elbow with WS (**A**: x 60, **B**: x 200). Crystalline substance in luminal structures under the dermis (red squares). This crystalline substance was examined with energy dispersive X-ray (EDX) (**C**, **D**). Each color and element combination corresponds to C for yellow, O for orange, Na for light blue, Mg for pink, Si for green, P for blue, and Ca for red. Using EDX, we measured the calcium content of the luminal structure, dermal connective tissue, and non-tissue areas at three points in each area (yellow circles), and performed similar measurements in three fields (**E**). Comparison of the calcium content of the luminal structure, dermal connective tissue, and non-tissue areas (**F**). Data are expressed as mean ± standard error. One-way ANOVA followed by Tukey test were performed (* p < 0.01, **** p < 0.0001).

**Figure 3:**
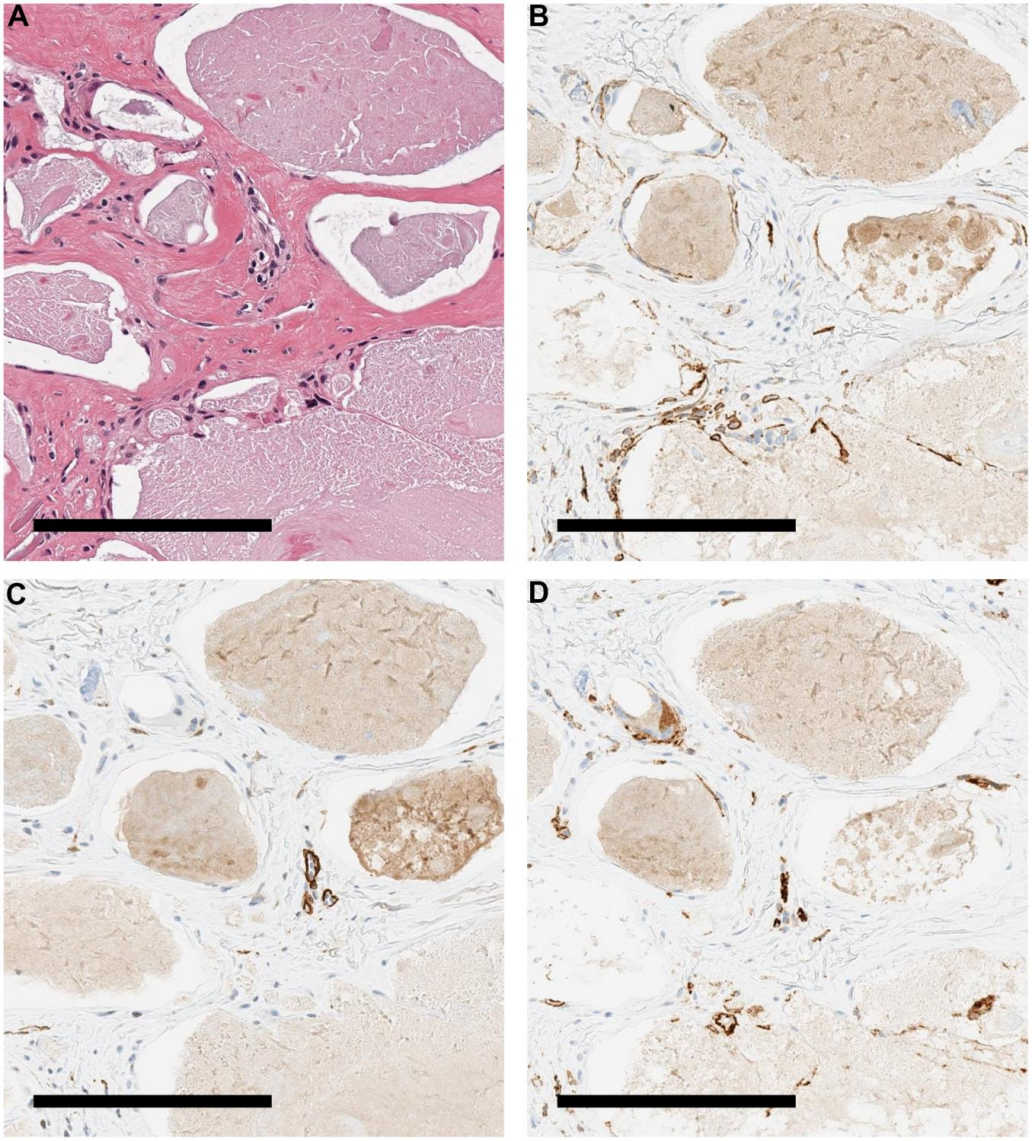
**Immunohistochemical staining image of the patient's skin.** Various stained tissue images of calcified tissues from a patient with WS. (**A**) HE. (**B**) Immunohistochemical staining with podoplanin antibody. Cells around the calcification were positive. (**C**) Immunohistochemical staining with αSMA antibody. Cells around the calcification were negative. (**D**) Immunohistochemical staining with CD 31 antibody. The cells around the calcification were weakly positive. All scale bars are 200 μm.

Radiographs of four patients with WS who had ulcers on the elbow revealed calcification in the subcutaneous tissue of the ulcerated elbow joint in all patients (Figure 1A, 1B). In skin tissue taken from the same ulcers, calcification was present in three of the four patients (Figure 1C, 1D). We observed the skin tissue of a patient with WS (WS2) with calcification of the skin by scanning electron microscopy. There was a deposition of crystalline substances in the luminal structure under the dermis (Figure 2A, 2B). Next, we performed energy dispersive X-ray (EDX) analysis of the crystalline substance (Figure 2C, 2D) and detected the characteristic X-rays of phosphorus and calcium with high counts suggestive that the crystalline substance was calcium phosphate. To determine whether tissue other than luminal structures contained calcium, we compared calcium content in luminal structures, dermal connective tissues, and areas that did not contain tissue by EDX. We measured three points in each area and performed similar measurements in three fields (Figure 2E). One-way analysis of variance (ANOVA) was performed for the three groups, followed by Tukey’s test. The calcium content in the luminal structure was significantly higher than that in the dermal connective tissue (P<0.0001; mean difference 22101kev; CI=-18844 to -25359) and the calcium content in the dermis connective tissue was not significantly different from that in the non-tissue region (P<0.0001; mean difference -1985 μm2; CI=-2514 to -1456) (Figure 2F). Immunohistochemical staining was performed using podoplanin, CD31, and αSMA antibodies to identify the luminal structure in which the calcified material was deposited. The luminal structure around the calcification was positive for the D2 -40 antibody, weakly positive for the CD 31 antibody, and negative for the αSMA antibody. These results indicate that this luminal structure was a LEC (Figure 3A–3D).

### Increased lymphangiogenesis and impaired lymphatic vessel space development around ulcers in patients with WS

**Figure 4:**
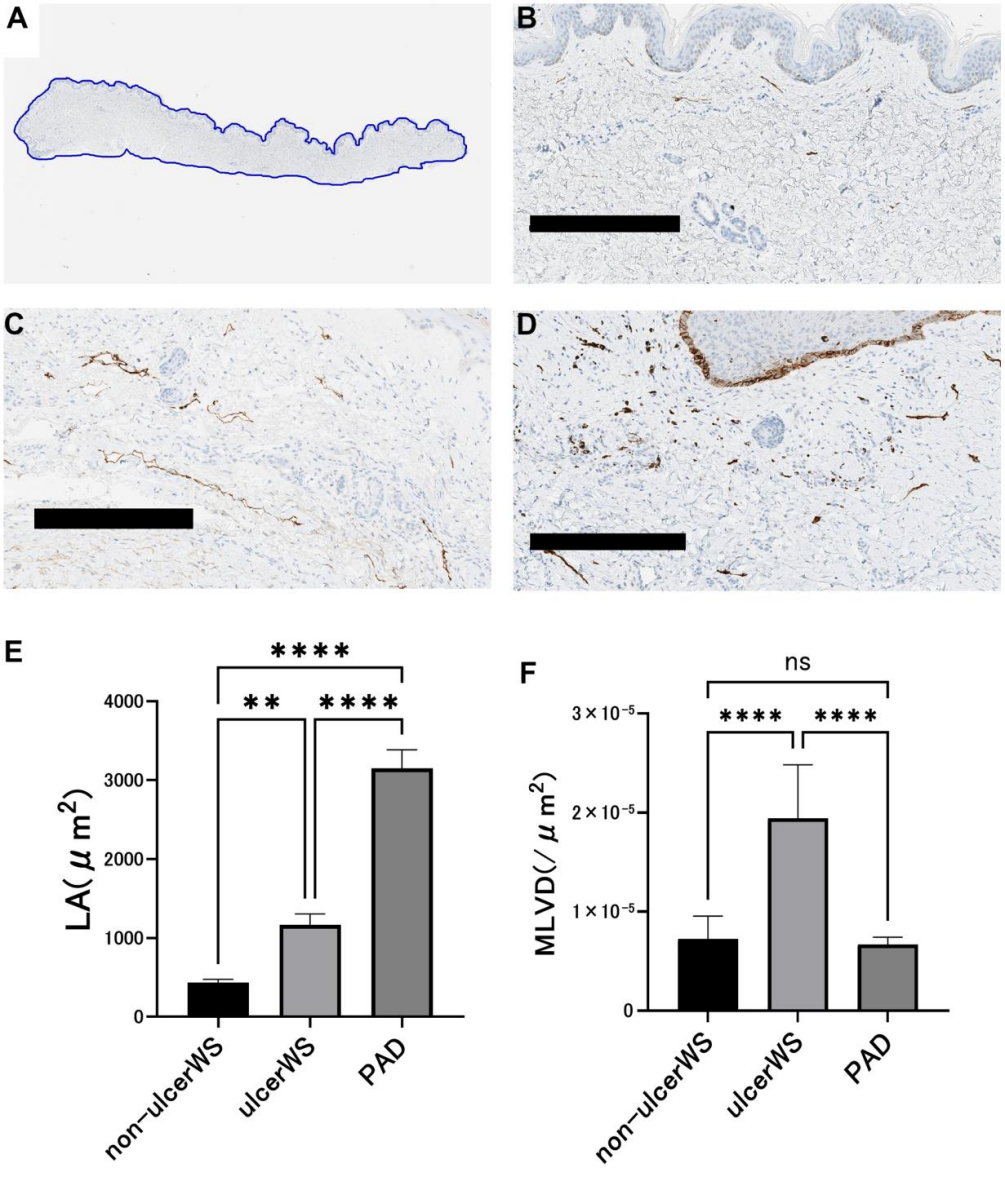
**Image analysis of lymphatic vessels in the skin of patients.** (**A**) The area of skin tissue sections was measured by tracing the skin margins using an Aperio Image Scope. (**B**) A representative example of skin histology taken from an ulcer-free area in a patient with Werner syndrome. Scale bar is 300 μm. The lumen is narrow and small in number. (**C**) A representative example of skin tissue taken from the ulcer circumference of a patient with PAD. Scale bar is 300 μm. The lumen is dilated and lined up. (**D**) A representative example of skin tissue taken from the ulcer circumference of a Werner syndrome patient. Scale bar is 300 μm. The lumen is indistinct and randomly proliferating. (**E**) Comparison of luminal cross-sectional area (LA) of the lymphatic vessels. In four patients with Werner syndrome, two different sites were measured in the non-ulcerated area and in the skin surrounding the ulcer. Three patients with PAD underwent measurements at two different sites of skin around the ulcer. Data are expressed as mean ± standard error. One-way ANOVA followed by Tukey test were performed (* * p < 0.001, **** p < 0.0001). (**F**) Comparison of lymphatic vessel density (MLVD). In four patients with Werner syndrome, two different sites were measured in the non-ulcerated area and in the ulcer's skin. Three patients with PAD underwent measurements at two different sites of skin around the ulcer. Data are expressed as mean ± standard error. One-way ANOVA followed by Tukey test were performed (n.s. No significant difference, **** p < 0.0001).

To clarify the histological features of lymphatic vessels in patients with WS, we compared immunohistochemical staining using the podoplanin antibody in the skin of the non-ulcerated area (non-ulcer WS group) and the skin around the ulcer (ulcer WS group) of four patients with WS and the skin around the foot ulcer (ulcer PAD group) of three patients with peripheral arterial disease (PAD). In the non-ulcer WS group, the lymphatic vessels were poorly endoluminal and sparse in density. In the ulcer WS group, lymphatic vessels with poorly developed lumens proliferated in an irregular arrangement. In the ulcer PAD group, the lymphatic lumina expanded and aligned linearly (Figure 4B–4D). We measured the intradermal luminal cross-sectional area (LA) of the lymphatic vessels in the three groups (Table 1). One-way analysis of variance (ANOVA) was performed for the three groups, followed by Tukey’s test. LA in the ulcer WS group was larger than in the non-ulcer WS group (P=0.0034; mean difference -730.1 μm^2^; CI=-1220 to -240.0) and smaller than in the ulcer PAD group (P<0.0001; mean difference -1985 μm^2^; CI=-2514 to -1456) (Figure 4E). Microlymphatic vessel density (MLVD) was measured in all three groups (Table 1). According to Tukey test, post one-way ANOVA, MLVD in the ulcer WS group was higher than in the non-ulcer WS (P<0.0001; mean difference -1.21x10^-5^/μm^2^; CI=-1.67x10^-^5 to -7.60x10^-6^) and ulcer PAD groups (P<0.0001; mean difference 1.27x10^-5^/μm^2^; CI=7.80x10^-6^ to 1.766x10^-5^) (Figure 4F).

**Table 1:** LA and MVLD.

**Group**	**N**	**LA (μm^2^)**		**MVLD (/μm^2^)**	
		**Mean**	**SE**	**Mean**	**SE**
non-ulcer WS	8	434.7	40.25	7.24x10^-6^	8.15x10^-7^
ulcer WS	8	1165	139.3	1.94x10^-5^	1.91x10^-6^
ulcer PAD	6	3150	234.5	6.68x10^-6^	3.04x10^-7^

LA, luminal cross-sectional area; MLVD, lymphatic microvessel density; WS, Werner syndrome; SEM, standard error of the mean.

### In LECs of WS, WRN protein accumulates in the cytoplasm

**Figure 5:**
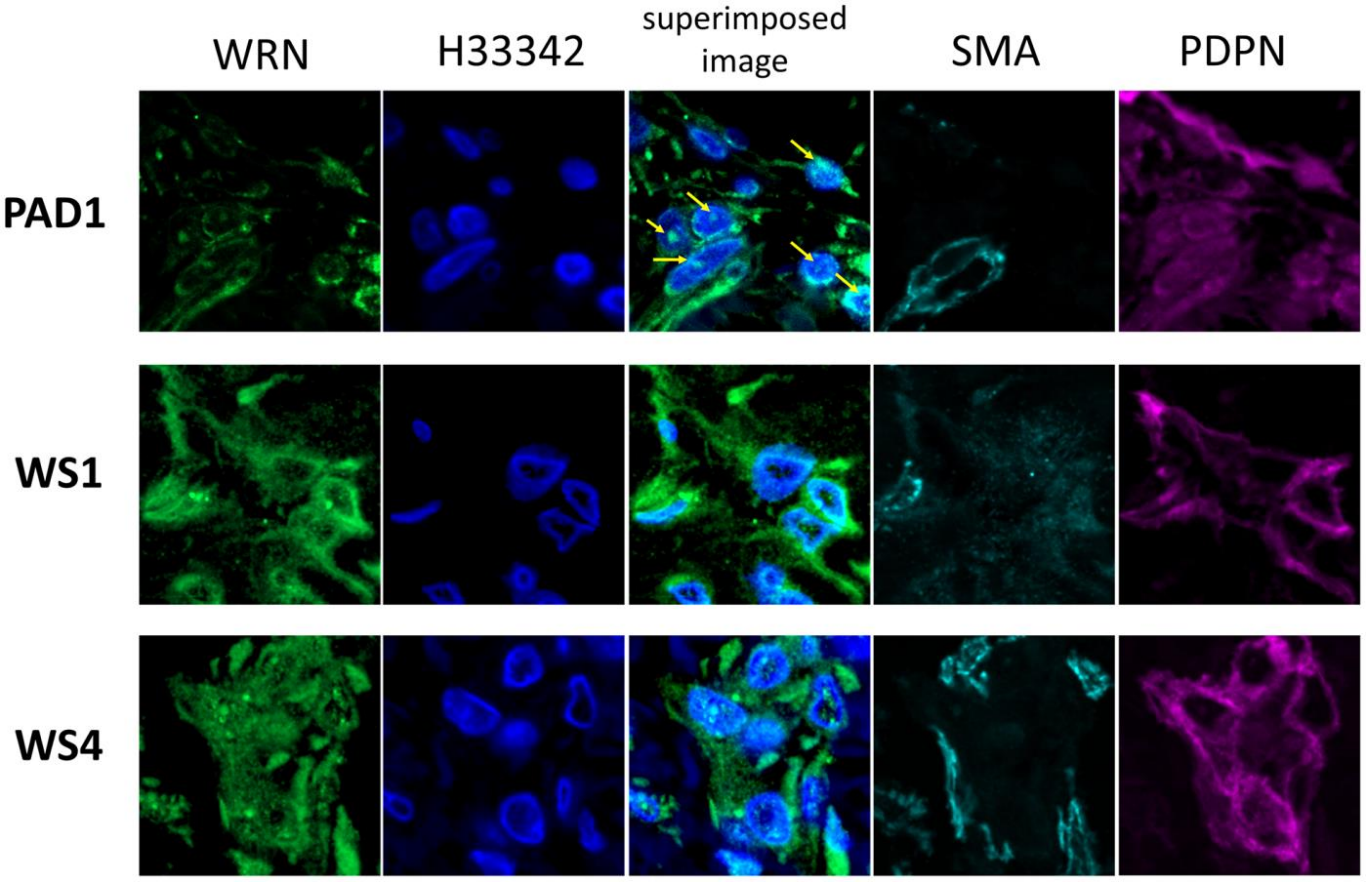
**Immunofluorescent staining images of lymphatic vessels of the patient's skin.** Immunofluorescent staining images of lymphatic vessels in the dermis of two patients with WS and one with PAD are shown. A stained image with WRN antibody, Hoechst 33342, superimposed image, αSMA antibody, and podoplanin antibody are shown on the left. Samples collected from PAD1, WS1, and WS4 are shown at the top. In PAD1, the WRN protein is localized in the nucleus (arrow), whereas in WS1 and WS4, it is spread throughout the cytoplasm.

We investigated the subcellular localization of WRN protein in lymphatic endothelial cells of ulcerated skin tissue of 2 WS patients (WS1, WS4) and 1 PAD patient (PAD1) using a confocal microscope (Figure 5). In PAD patients, WRN protein is mainly present in the perinuclear lesion and shows nucleolus-like staining in nuclear. Conversely, the expression in WS1 and WS4 diffusely increased and distributed through the cytoplasm of the podoplanin positive cells identified as LECs. LECs with the increased WRN protein in the cytoplasm appeared in the regenerative LECs consisting of the lymphatic vessels with structural abnormalities. Moreover, WS4 showed the increased WRN protein in the cytoplasm and the nucleus. In conclusion, mutated WRN protein accumulation in the cytoplasm may associate with the developing abnormalities in the lymphatic vessels. One of the plausible mechanisms is that the increased accumulation of WRN in the cytoplasm causes the degeneration and the abnormal remodeling of lymphatic vessels with aging in WS patients, resulting in abnormalities in the lymphatic vessels and the developed luminal crystalloids through the impaired drainage function.

## DISCUSSION

This study reports that the subcutaneous calcification of WS grows in lymphatic vessels, and the crystalloids materials in luminal grow with the continuing disorders on lymphatic vessels related to aging. Moreover, it reveals the characteristic features of the subcutaneous calcification of WS patients that the lymphatic endothelial cells stored the WRN mutant protein in the cytoplasm and formed narrower vessels than them in the control group. Furthermore, it is noteworthy that lymphatic vessels are of the mesoderm origin. Because age-related disorders of WS generally occur predominantly on mesodermal organs such as bones and arteries, the subcutaneous calcification of WS can be regarded as the age-related changes in WS patients [14].

First, we performed a histological and ultrastructural analysis of soft tissue calcification appearing in WS and observed that crystalline substances consisting mainly of calcium phosphate were distributed exclusively in expanded lymphatic vessels located deep in the subcutaneous tissue. The crystalline material was deposited between the dermis and subcutaneous tissue, with prominent lymphangiogenesis of the small luminal structures. A previous report indicated that cultured dermal fibroblasts from WS deposit calcium *in vitro* [13]. Similarly, it is inferred that calcium phosphate is overproduced by WS fibroblasts, even *in vivo*.

In this study, we revealed that the site of subcutaneous calcification in WS patients is lymphatic vessels. Conversely, it has been considered that fibroblast ossification forms subcutaneous calcification of WS patients. Because calcification occurred on the *in vitro* cultured skin fibroblasts from WS patients, and the cells exhibited a higher bone-inducing ability *in vitro* assays [13, 15]. In contrast, this study demonstrated that the crystalloids materials exclusively exist in lymphatic vessels using quantitative elemental analysis for the subcutaneous calcification from WS patients. Moreover, we detected no significant increase in the amount of calcium element in the stromal tissue surrounding the lymphatic vessels. Therefore, generally, we concluded that lymphatic vessels are a place where calcified crystalloids grow in WS patients.

PAD is a specific disease that forms ulcers in the extremities of older adults and has characteristics of arteriosclerotic calcification. Therefore, we used PAD as a control group to clarify the characteristics of WS. There was no evidence of lymphatic calcification in the ulcerated area of PAD, suggesting that lymphatic calcification may be involved in the specific mechanism of WS.

The other characteristic findings in subcutaneous calcification of WS patients is the decreased diameter of lymphatic vessels, which can refer to abnormal aging in WS. In support of this hypothesis, it has been pointed out that decreased diameter and drainage function in lymphatic vessels are typical age-related changes [16, 17]. In this respect, WS needs to link with lymphatic changes as an age-related disease, whereas PAD must correlate with arteriosclerotic calcification.

It is noteworthy that calcification in the lymphatic vessels appears in only the ulcerative tissue and that the density was increased. This is not observed in non-ulcerated areas and is a distinct feature of typical age-related changes, suggesting the presence of unique factors in WS ulcerated areas. In this regard, studies comparing skin fibroblasts from the trunk of WS patients with those from lower extremity limbs have shown that lower extremity fibroblasts strongly express genes related to bone formation. Differences in preload of calcium phosphate to lymphatic vessels may account for differences in findings by site [15]. Furthermore, not all tissues are generally aged in WS. Responses to inflammatory lymphangiogenesis signals may be preserved compared with ordinary older adults, and further studies are needed to clarify differences in lymphatic vessel density.

This study found that LECs of WS patients showed accumulation of WRN protein diffusively distributed in the cytoplasm by confocal microscopic analysis. Moreover, we raised the possibility that mineralization in the lymphatic vessels links with abnormality in lymphatic endothelial cells with WRN accumulation. In this respect, we consider that the cytoplasmic accumulation of WRN proteins is one of the factors as aging in LECs, which causes the impaired functions of lymphatic vessels through the increasing cellular stresses, either supplying or draining interstitial fluid and metabolites.

Although, to date, various types of mutations in the WRN gene are in WS patients, while mutated proteins generally lack nuclear localization signals [2, 18–20]. Supporting this hypothesis, earlier studies using knock-in mice have shown that WRN mutant proteins accumulated in the cytoplasm of WRN mutant mice cause oxidative stress in the cells, resulting in various symptoms associated with aging [21–23]. However, the phenotype in WS patients does not appear in the knock-out mice lacking the WRN gene.

Therefore, it would be necessary for aging research to study the mechanisms between the lymphatic vessels and the cellular stress derived from the cytoplasmic accumulation of the mutated WRN protein. As there was no difference in clinical symptoms between patients with/without nuclear localization of WRN (WS1 and WS4), the prognostic roles on the diminished WRN protein in nuclear need to be elucidated in further study.

There are some limitations to this study. First, we did not use normal adult skin as a control instead, we initially used skin tissue discarded during surgery to treat ulcers. However, it is expected that there is a difference even among healthy individuals and WS patients because there was a significant difference in comparison with the PAD patient who did not have WS.

Next, this study clarified the pathology of skin calcification in WS histopathologically, and we have not yet elucidated its etiology. To explain the mechanism by which the accumulation of intracellular WRN mutant protein brings about the change of the lymph duct and what kind of difference of the clinical manifestation the degree of the accumulation brings about, further research in the future is necessary. Finally, the sample size in this study was small. Because Werner syndrome is a rare disorder, removing skin tissue for therapeutic purposes is even rarer, it is not easy to conduct extensive observational studies. Most of the research reports on Werner syndrome so far have only a few cases. We analyzed specimens from valuable surgical cases and identified cutaneous soft tissue calcification features, a specific symptom in 80% of Werner's syndrome. Whether or not the findings obtained in this study are universally present needs to be widely verified and confirmed in future studies.

In this study, we observed that immature lymphatic capillaries proliferated in the skin around ulcers in WS, and calcium phosphate accumulated in the lumen. In the cytoplasm of lymphatic endothelial cells of patients with WS, WRN mutant proteins accumulated at high concentrations. These results suggest that the accumulation of mutant proteins damages lymphatic endothelial cells, causing changes in lymphatic vessels similar to aging, resulting in reduced drainage function of lymphatic vessels in WS. This decreased lymphatic drainage function is thought to be one of the causes of subcutaneous tissue calcification, prolonged inflammation, and delayed wound healing. Our new findings have revealed part of the calcification, ulceration, and other conditions that plague many WS patients and could be a new step toward further research.

## MATERIALS AND METHODS

### Patient and control individuals

This study was approved by the Institutional Review Board of the Graduate School of Medicine, Chiba University (approval number: 973). The participants provided written informed consent before participating in the study.

We analyzed the skin tissues of patients with WS who developed painful ulcers at radiographically calcified sites (elbows and ankles) and required surgical treatment. The control group consisted of skin tissue from patients with chronic skin ulcers of the lower extremities associated with PAD and chronic renal failure. The profiles of the included patients are presented in Table 2. The types of WRN mutations in each patient with WS are listed in Table 3. These mutations resulted in the loss of the C-terminus after 1369-1402aa, where a nuclear localization signal was present. Mutation c. 3139 -1 G > C of WS2, 3, and 4 is the most common mutation in Japan [24].

**Table 2:** The profiles of the patients.

**Patient**	**Age**	**Sex**	**Location**	**Comorbidity**
WS1	62	M	elbow	DM, dyslipidemia, osteoporosis, CKD
WS2	48	M	elbow	DM, dyslipidemia, osteoporosis
WS3	50	F	elbow	DM, dyslipidemia, osteoporosis
WS4	57	M	elbow	DM, dyslipidemia, osteoporosis
PAD1	56	M	foot	DM, CKD
PAD2	71	M	foot	DM, CKD
PAD3	71	M	foot	CKD

WS, Werner syndrome; PAD, peripheral arterial disease; DM, diabetes mellitus; CKD, chronic kidney failure.

**Table 3:** WRN mutations in the patients.

**Patient**	**Nucleotide notation**	**Resulted codon**	**Resulted proteins (aa)**
WS1	homo c.1105C>T	Nonsense mutation	368
WS2	homo c.3139-1G>C	Frame shift/Nonsense mutation	1060
WS3	c.3913C>T c.3139-1 G>C	Nonsense mutation Frame shift/Nonsense mutation	1304 1060
WS4	c.3139-1 G>C c.3446 del T	Frame shift/Nonsense mutation Frame shift/Nonsense mutation	1060 1160

WS, Werner syndrome; aa, amino acid.

### Tissue sampling

In patients with WS, we sampled sclerotic skin around the ulcer that was removed simultaneously with debridement and normal skin that was used as a donor site for skin grafting. Resected specimens were fixed in 10% buffered formalin. In the PAD patient group, we sampled the skin at the boundary between the necrotic tissue and normal tissue from the amputated forefoot. All specimens were fixed in 10% buffered formalin and embedded in paraffin (FFPE tissues).

### Detection of crystalline substance using scanning electron microscopy

Scanning electron microscopy was used to map the distribution of crystalline substance in the tissue sections using an energy dispersive X-ray analyzer (SEM-EDX, TM4000Plus, Hitachi High-Technologies, Tokyo, Japan). Briefly, the 4 μm thick FFPE tissue sections were deparaffinized with xylene and washed well with 100% ethanol to eliminate wax and xylene remaining on the sections and dried. Then, well-dried sections were coated at 3 nm thickness with osmium using an osmium coater Neoc (Meiwafosis Co., Ltd., Japan) instrument and subjected to SEM-EDX analysis.

### Histological analysis

Histological analysis was performed on hematoxylin-eosin (H-E) stained 4um sections prepared from FFPE surgically resected skin tissues as previously described.

Immunohistochemical staining was performed using the Ventana XT system BenchMark (Ventana Medical Systems, Tucson, AZ, USA). Briefly, the tissue sections were automatically treated with an antigen retrieval solution (Ventana) and heated on a slide heater at 100° C for 30 min. Endogenous peroxidase activity was halted by immersion in 3% hydrogen peroxide for 4 min. The sections were then incubated with primary antibodies for 30 min at 37° C. According to the manufacturer's instructions, the LSAB Ventana Iview DAB detection system (Ventana) detects the immune complex and hematoxylin counterstained nuclei of 4um sections prepared from FFPE surgically resected tissues. Antibodies against podoplanin (D2-40, Ventana), smooth muscle actin (1A4, Ventana), and CD31(JC70, Ventana), respectively, were used to detect cytoplasmic expression in blood and lymphatic vessels, as previously reported [25].

### Image analysis

All image analyses were performed on digital slides (Leica Biosystems Aperio ImageScope v 12.4.7018). Microlymphatic vessel density (MLVD) in skin tissue was calculated by counting all D2 -40 positive lymphatic vessels and dividing by the skin area. The skin area and luminal cross-sectional area (LA) of the lymphatic vessels were measured by tracing the lymphatic vessels on a digital slide (Figure 4A). LA was calculated as the mean of the largest to the 10^th^ largest. To eliminate data variability, we performed measurements on two samples per case.

### Histological analysis through immunofluorescence detection

Monoclonal antibodies against α-WRN antibody (ab200, Abcam) were used to detect Werner's syndrome helicase protein expression. This antibody binds to the N-terminus of the WRN protein. In addition, both hoechst33342 (ThermoFisher Scientific, MA, USA) and anti-α-SMA antibody labeled with eFluor 660 (1A4, ThermoFisher Scientific) and anti-podoplanin labeled with Alexa Fluor 594 (BioLegend, CA, USA) were used to evaluate the cellular localization of Werner's syndrome helicase protein expression in lymphatic vessels with a confocal microscope (LSM710, Carl Zeiss).

### Statistical analyses

Data are presented as means ± SEM as indicated. One-way ANOVA followed by Tukey’s test was performed to compare the groups. Differences between groups were considered significant at p < 0.05. All tests were performed using GraphPad Prism 9.10 software (San Diego, CA, USA).

## References

[1] Epstein CJ, Martin GM, Schultz AL, Motulsky AG. Werner’s syndrome a review of its symptomatology, natural history, pathologic features, genetics and relationship to the natural aging process. Medicine (Baltimore). 1966; 45:177–221. 10.1097/00005792-196605000-00001 5327241

[2] Goto M, Tanimoto K, Horiuchi Y, Sasazuki T. Family analysis of Werner’s syndrome: a survey of 42 Japanese families with a review of the literature. Clin Genet. 1981; 19:8–15. 10.1111/j.1399-0004.1981.tb00660.x 7460386

[3] Yu CE, Oshima J, Fu YH, Wijsman EM, Hisama F, Alisch R, Matthews S, Nakura J, Miki T, Ouais S, Martin GM, Mulligan J, Schellenberg GD. Positional cloning of the Werner’s syndrome gene. Science. 1996; 272:258–62. 10.1126/science.272.5259.258 8602509

[4] Gray MD, Shen JC, Kamath-Loeb AS, Blank A, Sopher BL, Martin GM, Oshima J, Loeb LA. The Werner syndrome protein is a DNA helicase. Nat Genet. 1997; 17:100–3. 10.1038/ng0997-100 9288107

[5] Huang S, Li B, Gray MD, Oshima J, Mian IS, Campisi J. The premature ageing syndrome protein, WRN, is a 3'-->5' exonuclease. Nat Genet. 1998; 20:114–6. 10.1038/2410 9771700 PMC4940158

[6] Brosh RM Jr, Opresko PL, Bohr VA. Enzymatic mechanism of the WRN helicase/nuclease. Methods Enzymol. 2006; 409:52–85. 10.1016/S0076-6879(05)09004-X 16793395

[7] Rossi ML, Ghosh AK, Bohr VA. Roles of Werner syndrome protein in protection of genome integrity. DNA Repair (Amst). 2010; 9:331–44. 10.1016/j.dnarep.2009.12.011 20075015 PMC2827637

[8] Gaetani SA, Ferraris AM, D’Agosta A. Case report 485: Werner syndrome. Skeletal Radiol. 1988; 17:298–301. 10.1007/BF00401817 3212496

[9] Laroche M, Ricq G, Cantagrel A, Amigues JM, Mazieres B. Bone and joint involvement in adults with Werner’s syndrome. Rev Rhum Engl Ed. 1997; 64:843–6. 9476275

[10] Walton NP, Brammar TJ, Coleman NP. The musculoskeletal manifestations of Werner’s syndrome. J Bone Joint Surg Br. 2000; 82:885–8. 10.1302/0301-620x.82b6.10457 10990317

[11] Leone A, Costantini AM, Brigida R, Antoniol OM, Antonelli-Incalzi R, Bonomo L. Soft-tissue mineralization in Werner syndrome. Skeletal Radiol. 2005; 34:47–51. 10.1007/s00256-004-0792-8 15138723

[12] Taniguchi A, Tanaka Y, Takemoto M, Kubota Y, Taniguchi T, Motegi SI, Nakagami H, Maezawa Y, Koshizaka M, Kato H, Tsukamoto K, Mori S, Kuzuya M, Yokote K. Management guideline for Werner syndrome 2020 8. Calcification in tendons associated with Werner syndrome. Geriatr Gerontol Int. 2021; 21:163–5. 10.1111/ggi.14084 33260264

[13] Honjo S, Yokote K, Fujimoto M, Takemoto M, Kobayashi K, Maezawa Y, Shimoyama T, Satoh S, Koshizaka M, Takada A, Irisuna H, Saito Y. Clinical outcome and mechanism of soft tissue calcification in Werner syndrome. Rejuvenation Res. 2008; 11:809–19. 10.1089/rej.2007.0649 18729813

[14] Kudlow BA, Kennedy BK, Monnat RJ Jr. Werner and Hutchinson-Gilford progeria syndromes: mechanistic basis of human progeroid diseases. Nat Rev Mol Cell Biol. 2007; 8:394–404. 10.1038/nrm2161 17450177

[15] Kato H, Maezawa Y, Takayama N, Ouchi Y, Kaneko H, Kinoshita D, Takada-Watanabe A, Oshima M, Koshizaka M, Ogata H, Kubota Y, Mitsukawa N, Eto K, et al. Fibroblasts from different body parts exhibit distinct phenotypes in adult progeria Werner syndrome. Aging (Albany NY). 2021; 13:4946–61. 10.18632/aging.202696 33627520 PMC7950285

[16] Karaman S, Buschle D, Luciani P, Leroux JC, Detmar M, Proulx ST. Decline of lymphatic vessel density and function in murine skin during aging. Angiogenesis. 2015; 18:489–98. 10.1007/s10456-015-9479-0 26260189

[17] Jakic B, Kerjaschki D, Wick G. Lymphatic Capillaries in Aging. Gerontology. 2020; 66:419–26. 10.1159/000508459 32580201

[18] Suzuki T, Shiratori M, Furuichi Y, Matsumoto T. Diverged nuclear localization of Werner helicase in human and mouse cells. Oncogene. 2001; 20:2551–8. 10.1038/sj.onc.1204344 11420665

[19] Matsumoto T, Imamura O, Yamabe Y, Kuromitsu J, Tokutake Y, Shimamoto A, Suzuki N, Satoh M, Kitao S, Ichikawa K, Kataoka H, Sugawara K, Thomas W, et al. Mutation and haplotype analyses of the Werner’s syndrome gene based on its genomic structure: genetic epidemiology in the Japanese population. Hum Genet. 1997; 100:123–30. 10.1007/s004390050477 9225981

[20] Friedrich K, Lee L, Leistritz DF, Nürnberg G, Saha B, Hisama FM, Eyman DK, Lessel D, Nürnberg P, Li C, Garcia-F-Villalta MJ, Kets CM, Schmidtke J, et al. WRN mutations in Werner syndrome patients: genomic rearrangements, unusual intronic mutations and ethnic-specific alterations. Hum Genet. 2010; 128:103–11. 10.1007/s00439-010-0832-5 20443122 PMC4686336

[21] Aumailley L, Garand C, Dubois MJ, Johnson FB, Marette A, Lebel M. Metabolic and Phenotypic Differences between Mice Producing a Werner Syndrome Helicase Mutant Protein and Wrn Null Mice. PLoS One. 2015; 10:e0140292. 10.1371/journal.pone.0140292 26447695 PMC4598085

[22] Aumailley L, Dubois MJ, Garand C, Marette A, Lebel M. Impact of vitamin C on the cardiometabolic and inflammatory profiles of mice lacking a functional Werner syndrome protein helicase. Exp Gerontol. 2015; 72:192–203. 10.1016/j.exger.2015.10.012 26521679

[23] Aumailley L, Dubois MJ, Brennan TA, Garand C, Paquet ER, Pignolo RJ, Marette A, Lebel M. Serum vitamin C levels modulate the lifespan and endoplasmic reticulum stress response pathways in mice synthesizing a nonfunctional mutant WRN protein. FASEB J. 2018; 32:3623–40. 10.1096/fj.201701176R 29452565 PMC5998970

[24] Huang S, Lee L, Hanson NB, Lenaerts C, Hoehn H, Poot M, Rubin CD, Chen DF, Yang CC, Juch H, Dorn T, Spiegel R, Oral EA, et al. The spectrum of WRN mutations in Werner syndrome patients. Hum Mutat. 2006; 27:558–67. 10.1002/humu.20337 16673358 PMC1868417

[25] Nakagawa A, Fujimoto H, Nagashima T, Sangai T, Takada M, Masuda T, Teranaka R, Ota S, Matsushima J, Akita S, Ohtsuka M. Histological features of skin and subcutaneous tissue in patients with breast cancer who have received neoadjuvant chemotherapy and their relationship to post-treatment edema. Breast Cancer. 2020; 27:77–84. 10.1007/s12282-019-00996-x 31346921

